# Procalcitonin and High APACHE (Acute Physiological and Chronic Health Evaluation) Level Are Associated with the Course of Acute Kidney Injury in Patients with SARS-CoV-2

**DOI:** 10.1155/2022/1363994

**Published:** 2022-10-07

**Authors:** Jorge Andrade Sierra, Claudia Delgado Astorga, Miriam Gabriela Nava Vargas, Enrique Rojas Campos, Kevin Javier Arrelano Arteaga, Karla Hernández Morales, Carlos A. Andrade Castellanos, Antonio de Jesús Andrade-Ortega, Luis Gerardo González Correa

**Affiliations:** ^1^Department of Internal Medicine, Hospital Civil de Guadalajara “Dr. Juan I. Menchaca”, Guadalajara, Jalisco, Mexico; ^2^Department of Physiology, University Health Sciences Center, University of Guadalajara, Guadalajara, Jalisco, Mexico; ^3^Medical Research Unit in Kidney Diseases, Specialties Hospital, National Western Medical Center, Mexican Institute of Social Security, Guadalajara, Jalisco, Mexico

## Abstract

**Background:**

Acute kidney injury (AKI) is associated with poor outcomes in patients infected with SARS-CoV-2. Sepsis, direct injury to kidney cells by the virus, and severe systemic inflammation are mechanisms implicated in its development. We investigated the association between inflammatory markers (C-reactive protein, procalcitonin, D-dimer, lactate dehydrogenase, and ferritin) in patients infected with SARS-CoV-2 and the development of AKI.

**Methods:**

A prospective cohort study performed at the Civil Hospital (Dr. Juan I. Menchaca) Guadalajara, Mexico, included patients aged >18 years with a diagnosis of SARS-CoV-2 pneumonia confirmed by RT-PCR and who did or did not present with AKI (KDIGO) while hospitalized. Biomarkers of inflammation were recorded, and kidney function was estimated using the CKD-EPI formula.

**Results:**

291 patients were included (68% males; average age, 57 years). The incidence of AKI was 40.5% (118 patients); 21% developed stage 1 AKI, 6% developed stage 2 AKI, and 14% developed stage 3 AKI. The development of AKI was associated with higher phosphate (*p* = 0.002) (RR 1.39, CI 95% 1.13–1.72), high procalcitonin levels at hospital admission (*p* = 0.005) (RR 2.09, CI 95% 1.26–3.50), and high APACHE scores (*p* = 0.011) (RR 2.0, CI 95% 1.17–3.40). The survival analysis free of AKI according to procalcitonin levels and APACHE scores demonstrated a lower survival in patients with procalcitonin >0.5 ng/ml (*p* = 0.001) and APACHE >15 points (*p* = 0.004).

**Conclusions:**

Phosphate, high procalcitonin levels, and APACHE levels >15 were predictors of AKI development in patients hospitalized with COVID-19.

## 1. Introduction

The coronavirus disease 2019 (COVID-19) pandemic, which originated with the novel coronavirus (SARS-CoV-2), caused 318 thousand deaths in Mexico as of February 2022 [1]. Older adults with comorbidities [2, 3] are at a higher risk of complications from SARS-CoV-2 infections. Kidney damage is one of the main complications and can be demonstrated by the presence of hematuria, proteinuria [4–6], and the development of acute kidney injury (AKI), with a high incidence reported in hospitalized patients. [7–10] The severity leads to even higher mortality, which has a multifactorial etiology [11, 12]. Nevertheless, baseline characteristics, patient interventions in critical care, and organ crosstalk are mechanisms that influence the appearance of AKI, but possible direct injury by the virus on kidney cells (podocytes, proximal tubule cells, and the epithelial cells of Bowman's capsule) [13–15] and uncontrolled systemic inflammation are factors that also influence its development and severity in patients with COVID-19 [4, 16, 17]. Inflammation and the consequent increase in some biomarkers also appear to be associated with kidney damage and poor outcomes [2, 3, 18–25]. In fact, the therapeutic conduct for the prevention of developing AKI associated with COVID-19 is similar to other etiologies (avoiding nephrotoxins, periodic review of serum creatinine (SCr), urinary output, and hemodynamic monitoring), and in critically ill patients with COVID-19, it could reduce the appearance or severity of AKI [26]. Our hospital has been a referral center for patients without social security throughout the pandemic, and the limitations of some resources, such as urinary markers (neutrophil gelatinase-associated lipocalin (NGAL) and kidney injury molecule 1 (KIM-1)), demand the use of low-cost inflammatory markers to predict the development of AKI. Our objective was to determine the existing association between CRP, PCT, D-dimer, LDH, and ferritin levels and the development and severity of AKI in patients hospitalized with pneumonia caused by SARS-CoV-2 without social security. In healthy individuals, PCT is produced in the thyroid *C* cells and is quickly converted to calcitonin, so levels of serum PCT are very low (<0.02 ng/ml), but in bacterial infections in many extrathyroidal tissues (kidney, spleen, adipocytes, pancreas, colon, brain, and lungs), PCT is synthesized yet such parenchymal tissue lacks the processing pathway necessary to convert PCT to calcitonin, thereby increasing PCT levels. [27] In viral infections, the production of interferon-gamma (IFN-g) in virus-infected cells inhibits the production of PCT, so theoretically it does not rise, but several studies show that increased PCT levels are positively associated with a higher risk of severity of COVID-19, and this increase could be dependent on the severity of the disease or associated with severe coinfection. [28, 29] A SARS-CoV-2 infection causes local and systemic inflammation mediated by proinflammatory cytokines. IL-1 stimulates the secretion of TNF, IL-6, and other cytokines, in a proinflammatory complex that can lead to a cytokine storm leading to lung and systemic impairment, and the release of PCT has also been associated with proinflammatory cytokines [28, 30].

## 2. Methods

A prospective cohort study was performed on patients without social security coverage (at the Internal Medicine Department of “Dr. Juan I. Menchaca” Civic Hospital in Guadalajara) from March 1, 2020, to March 1, 2021. All patients tested positive for SARS-CoV-2 using reverse transcriptase polymerase chain reaction (RT-PCR). Based on the development of AKI using the Kidney Disease Improving Global Outcomes (KDIGO) classification, patients were classified into two groups: those with and those without AKI. All patients in our study had normal kidney function when admitted to the hospital, and we could not clinically determine infections other than COVID-19.

### 2.1. Data Collection

Quantitative polymerase chain reaction (RT-PCR) was used to determine the presence of SARS-CoV-2 in the nasopharyngeal swabs. All registered patients were tested for inflammatory biomarkers (lactate dehydrogenase, ferritin, highly sensitive C-reactive protein, D-dimer, and procalcitonin) upon admission and during hospitalization, and the following data were collected: age, sex, history of smoking, alcoholism, prescribed medication use, and comorbidities. The patients' anthropometric characteristics and the presence of other infections (as documented by clinical evaluation or confirmed by any microbiological method) were also recorded. The following biochemical variables were recorded: glucose, urea, SCr, uric acid, glycated hemoglobin, lipids, liver enzymes, electrolytes, hemoglobin, platelets, leukocytes, and lymphocytes. Imaging studies, such as chest radiography or tomography, were performed on all patients upon hospital admission. Disease severity was assessed using the Acute Physiological and Chronic Health Evaluation II (APACHE II) scoring system and the Sequential Organ Failure Assessment (SOFA). Glomerular filtration rate (eGFR) was estimated using the CKD-EPI formula [31].

Patients with chronic kidney disease (CKD), kidney transplant, obstructive kidney comorbidities, single kidney, neoplastic or autoimmune diseases, chronic use of antiinflammatory drugs, and use of immunosuppressant drugs were excluded from the study.

### 2.2. Treatment Protocol in Patients with COVID-19 on Admission Hospital

All patients admitted to the hospital were managed with isolation measures and supplemental oxygen. According to the evolution of each patient, oxygen was escalated to high-flow nasal cannula oxygen, noninvasive ventilation, or invasive ventilation.

Dexamethasone at a dosage of 6 mg/day for 10 days [32] was administered to all those requiring supplemental oxygen, and thromboprophylaxis with enoxaparin was used at doses of 40 to 60 mg daily if there was no contraindication for its use.

Antibiotics were only used in cases of clinical or microbiological suspicion of bacterial coinfection and the most commonly used antibiotics were carbapenems, piperacillin/tazobactam, and vancomycin adjusted to GFR.

Fluid administration was adjusted in balance according to volume responsiveness and tolerance assessment, and the use of diuretics was based on clinical criteria. Critically ill patients with severe acute renal failure were treated with intermittent hemodialysis, applied daily if necessary, or peritoneal dialysis, but our center does not use continuous renal replacement therapy (CRRT) due to a lack of resources.

The present study complies with the ethical principles for medical research in human beings as stipulated by the Declaration of Helsinki, 64th General Assembly, Fortaleza, Brazil, October 2013, in addition to adhering to the standards of good clinical practice. All procedures were performed according to the national regulations stipulated in the General Health Legal Guidelines for Health Care Research in Mexico, 2nd Title, Ethical Aspects for Research in Human Beings, Chapter 1, Article 17. The study protocol was reviewed and approved by the Ethics Committee of the Hospital Civil de Guadalajara, “Dr. Juan I Menchaca” (*HCG-JIM*). Registration number: 17CI14 039 116 COFEPRIS. Guadalajara, Jalisco, Mexico.

## 3. Consent to Participate Statement

All patients signed informed consent forms prior to hospital admission and provided consent to participate in the study.

### 3.1. Inflammatory Markers

 
*Procalcitonin*. Measurements were performed using the sandwich principle of an automated electrochemiluminescent immunoassay (Cobas E411, Roche)
 
*Ferritin*. Measurements were performed using the immunoturbidimetric assay, Image 800 (days)
 
*Highly Sensitive C-Reactive Protein (hsCRP).* Measurements were performed using a particle-enhanced immunoturbidimetric assay Cobas® c501 (Roche)
 
*D-Dimer*. Measurements were performed using an immunochromatography assay: Ramp®

### 3.2. Definitions

#### 3.2.1. SARS-CoV-2 Pneumonia

This is defined by the National Institutes of Health as individuals with SARS-CoV-2 infection confirmed by RT-PCR testing who have SpO_2_ < 94% on room air at sea level, a ratio of arterial partial pressure of oxygen to fraction of inspired oxygen (PaO_2_/FiO_2_) < 300 mmHg, respiratory frequency >30 breaths/min, or lung infiltrates >50% [33].

#### 3.2.2. Acute Kidney Injury (AKI)

This is [34] an abrupt decrease in GFR is manifested by an increase in SCr or oliguria within the first 48 hours to seven days.

We classified AKI, considering only the increase in SCr:

  AKI1: increase in SCr 1.5 to 1.9 times the baseline level, or an increase of >0.3 mg/dl
  AKI2: increase in SCr 2 to 2.9 times the baseline level
  AKI3: increase in SCr by 3 times the baseline, an increase of SCr >4 mg/dl, or the onset of renal replacement therapy

### 3.3. Statistical Analysis

The data are presented as mean ± standard deviation or median, numbers, and percentages where appropriate. Student's *t*-test or the Mann–Whitney *U* test, depending on the distribution, was used to compare groups. The Cox proportional hazards model was used to estimate the risk of AKI. Survival free of AKI and mortality was evaluated using the Kaplan–Meier test. Statistical analysis was performed using SPSS™ software, version 17. A value of *p* < 0.05 was considered statistically significant.

## 4. Results

A total of 291 patients were eligible for inclusion in this study. The average age was 57 ± 14 years, and 68% (*n* = 198) corresponded to the male sex. At the time of hospital admission, the average body mass index (BMI) was 31 kg/m^2^, and approximately 40% of the patients had an associated comorbidity (DM or SAH). Ninety-five percent (95%) of patients required some form of supplementary oxygen, and 20% were categorized as having severe illness requiring high-flow nasal cannulas and/or invasive mechanical ventilation (IMV), with an average of 9.5 days of hospitalization. At admission, no patient clinically presented infectious processes other than COVID-19, and all patients received dexamethasone at a dosage of 6 mg/day for 10 days during their hospital stay. The demographic characteristics of patients with AKI and COVID-19 are presented in Table 1.

**Figure 1:**
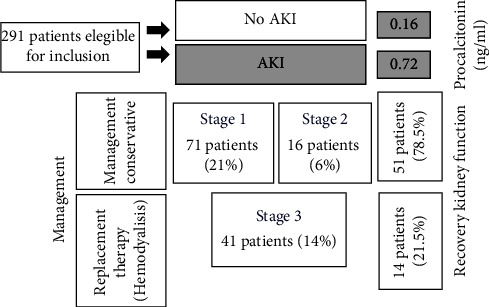
Clinical course, management, and recovery of the kidney function in patients who developed AKI with COVID-19.

Acute kidney injury (AKI): AKI was recorded in 40.5% (118 of the 291 patients). Seventy-one patients (21%) had stage 1 AKI, 16 (6%) had stage 2 AKI, and 41 (14%) had stage 3 AKI(Figure 1).

The development of severe sepsis or septic shock during follow-up was documented in 49% (58 of 118 patients) (*p* < 0.05). In the group that developed AKI, the male sex predominated (75% vs. 64% in the group without AKI, respectively) older age (61 vs. 54 years) and prolonged hospital stay (12 vs. 7.7 days) (*p* < 0.05). Approximately 50% of patients with AKI had some type of associated comorbidity (DM or SAH) compared to only 30% in the group without AKI (*p* < 0.05). The use of dexamethasone, angiotensin-converting enzyme inhibitors (ACEi), and angiotensin receptor blockers (ARBs) were similar in both groups, whereas the use of diuretics was higher in patients with AKI. The highest points in severity on the APACHE II and SOFA scores at the time of hospital admission were recorded in patients who developed AKI (*p* < 0.05). Baseline hemoglobin, platelets, leukocytes, lymphocytes, CRP, LDH, and ferritin levels at hospital admission did not differ between groups, while glucose levels (215 vs. 161), PCT (0.72 vs. 0.16), and D-dimer (670 mg/dL vs. 327 mg/dL) (*p*=0.001) were significantly higher in patients with AKI than those without AKI. During follow-up, inflammatory markers in AKI stages 2 and 3 presented higher levels of PCT on days 6 and 12 (*p*=0.009 and *p*=0.012, respectively), ferritin on days 6 and 12 (*p*=NS), and D-dimer on day 6 (*p*=0.017) (Table 2).

In the multivariate analysis, higher phosphate (*p*=0.002, RR 1.39, CI 95% 1.13–1.72), PCT level >0.5 ng/ml on hospitalization (*p*=0.005*, RR 2.09, CI 95% 1.26*–*3.50*), and >15 points on APACHE (*p*=0.011*; RR 2.0 CI 95% 1.17*–*3.40*) showed a significant association with the development of AKI (Table 3).

**Figure 2:**
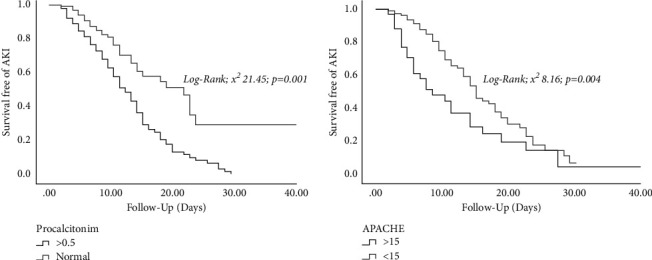
Acute kidney injury-free time according to procalcitonin and APACHE level.

Fifty-five percent of those who developed AKI recovered kidney function, the majority of whom had AKI stage 1. The survival analysis free of AKI according to PCT levels (≥0.5 ng/ml) (*p*=0.001) and points on the APACHE level (>15 points) (*p*=0.004) demonstrated a lower survival (Figure 2).

## 5. Discussion

The present study concurs with the Latin American registry [35] which describes a high frequency of AKI (65%). In Mexico, incidences of 33.7 and 58.6% are reported, where half correspond to severe cases, with comorbidities consistent with the present study, where we documented that 30% of patients were overweight or had some degree of obesity. [36, 37] The development of AKI has been associated not only with obesity but also with being overweight [38]. The present study did not perform an analysis of the obesity subgroups, but both study groups had similar BMIs, and there was no demonstrated association with the development of AKI. We found a high prevalence of SAH and DM with higher concentrations of glucose and HbA1C in those who developed AKI, but these were not associated with risk. Our results are consistent with the literature, where age is a risk factor for severe illness [19] and leads to a higher risk of AKI [39]. Hirsch et al. demonstrated a higher risk of AKI (*OR 1.03, CI 95% 1.03*–1.04; *p* < 0.001) in those aged >60 years [7]. Similarly, Fisher et al. [40] reported that older age (67 vs. 60 years; *p* < 0.001) was an independent risk factor for developing AKI. In Mexico, Casas-Aparicio et al. associated higher age with AKI development (*OR 1.07, CI 95% 1.01*–1.13; *p* = 0.024) [36]. In the present cohort, although patients in the AKI group were older (61 ± 14 vs. 54 ± 13; *p* < 0.001), we were unable to consolidate these results, not achieving statistical significance. Hyperphosphatemia is related to the risks of AKI and mortality [41]. In the present cohort, we did not find hyperphosphatemia, but phosphate levels were higher in the AKI group. There is evidence that AKI is a phenomenon associated with the activation of proinflammatory cytokines and therefore calls for the use of markers of kidney injury [27]. Some biomarkers predict AKI [42]. However, the high cost and lack of availability of these tests in our region forced us to use biochemical parameters more within reach in our practice in order to predict AKI events. Procalcitonin is barely detectable in healthy individuals; however, diverse conditions tend to elevate it (surgery, trauma, burns, pancreatitis, and sepsis) to levels that can be markedly high [43–47]. One of the advantages of PCT is its rapid increase of 6 h after the onset of the inflammatory process [27]. The elimination pathway of PCT is not clear, but due to its low molecular weight and the deterioration of kidney function, its levels can be elevated; therefore, it is not a reliable parameter in the case of illness because the thresholds are different [48–50]. Considering this aspect, the present study excluded patients with the onset of deterioration of kidney function as well as any patient who had conditions that would affect PCT values. The present multivariate analysis showed a significant risk for the development of AKI when PCT rates were >0.5 ng/ml at the moment of hospitalization. Some studies have demonstrated an association between PCT and the development of kidney damage in contexts other than COVID-19 [20, 43–45]. Feng et al. showed the sensitivity (76%) and specificity (75%) of PCT in predicting AKI (*OR 9.63, 95% CI, 95% 4.38–21.18*) [43], and Chun et al. [44] reported that in septic patients hospitalized in an intensive care unit (ICU), PCT was significantly high (*p* < 0.001) and was associated with AKI (OR 1.006, 1,000–1,011; *p* = 0,035). In the present study, 50% of the patients who developed AKI had a case of severe sepsis or septic shock that developed during their hospital stay (*p* < 0.05). Nevertheless, our analysis did not demonstrate an association of risk for the development of AKI. Regarding COVID-19, Wang et al. found that PCT levels were a predictor of kidney damage [51]. This association can be measured by an exaggerated inflammatory response, even if the pathophysiological mechanism is unclear [52, 53]. As a crucial component of the inflammatory cascade, the expression of PCT can be correlated with levels of systemic inflammation and the development of AKI in patients with COVID-19 [51]. Procalcitonin is a chemoattractant produced by monocytes in the inflammatory area, which contributes to the increase and recruitment of parenchymal cells with a greater release of cytokines associated with acute kidney damage [44]. Another implicated mechanism is the direct toxic effect of PCT on mesangial cells and apoptosis [53]. Additionally, other markers demonstrate increments dependent on the reduction of the GFT [54], and in AKI, the CRP and D-dimer levels have been shown to be high [36]. The reduction in kidney function can explain our results of the increase in D-dimer levels predominantly in AKI stages 2 and 3. However, the analysis did not show an association with the risk for AKI. In severely ill patients, the APACHE II score is used over the long term to predict hospital mortality up to 28 days in different contexts (sepsis and pancreatitis). Zou et al. documented excellent discriminative power for mortality in patients with COVID-19 with point scores ≥17 [55]. In the context of COVID-19, the APACHE II score was also associated with AKI in a Latin American cohort (OR 1.97, CI 95%, 1.08–2.64; *p* < 0.05), with higher mortality in those who had higher point scores (OR 1.08, CI 95%, 1.02–1.98; *p* < 0.05) and in those who developed stage 3 AKI (OR 1.11, CI 95%, 1.05–2.57; *p* < 0.05). These data support that in COVID-19 patients, AKI is a decisive phenomenon in the prognosis of these patients. In different studies on patients with COVID-19, higher APACHE II scores have been associated with poor prognosis and AKI severity [56, 57]. We found that >15 points on the APACHE II score demonstrated significance for the development of AKI, which could be explained by the severity of the clinical scenario of COVID-19 and the patient's comorbidities upon hospitalization. Until now, it remains unknown whether recovery from AKI in COVID-19 differs from other forms of AKI, and the direct, long-term impact of the SARS-CoV-2 virus on kidney function in those who regain function also remains unclear. Therefore, the recommendation is a follow-up period of at least 2 to 3 months [58]. Despite the high incidence of AKI, the majority of our patients had a favorable prognosis regarding recovery of kidney function since more than half of the patients studied had an improvement in GFR. However, progression from AKI stages 2 to 3 was associated with poor recovery.

## 6. Conclusions

In this retrospective cohort, significantly high PCT levels were found on hospital admission in patients who developed AKI, and this was a predictor of AKI development. The trajectory of PCT throughout the follow-up did not show significant differences between those who did and did not develop AKI. Therefore, the use of PCT reinforces the importance of this marker as a predictor for the development of AKI in patients with COVID-19 infection without superinfections when admitted to the hospital.

## 7. Limitations

The present study had some limitations. We recognize that this study is unicentric. Nevertheless, being a referral center for patients infected with SARS-CoV-2 who do not have social security in our region, favors extrapolation of the results to other populations, especially those with limited access to health care. The majority of patients before hospitalization had passed a considerable amount of time since acquiring the viral infection; therefore, subclinical bacterial superinfection could not be ruled out. Economic limitations (lack of treatment with continuous slow therapies) could influence the lack of recovery of kidney function, especially in those who developed severe AKI. Finally, the lack of measurement and/or comparison of markers valid for AKI did not permit the determination of PCT levels.

## 8. Disclosure

This manuscript was submitted as a preprint in the following link: https://www.medrxiv.org/content/10.1101/2022.08.09.22274874v1.

## Figures and Tables

**Table 1:** Demographic characteristics of patients with acute kidney injury (AKI) and COVID-19.

Characteristics	Total, *n* = 291	AKI, *n* = 118	No AKI, *n* = 173
Age (years)	57 ± 14	61 ± 14^*∗*^	54 ± 13^*∗*^
Sex-masculine, *n* (%)	198 (68)	88 (75)^*∗*^	110 (64)^*∗*^
Smoking, *n* (%)	89 (31)	41 (35)	48 (28)
Hospitalization (days)	9.5 ± 7.0	12 ± 7.8^*∗*^	7.7 ± 5.7^*∗*^

Comorbidities			
BMI, (Kg/m^2^)	30.9 ± 6.9	31.2 ± 7.5	30.6 ± 6.4
Diabetes, *n* (%)	113 (39)	55 (46.6)^*∗*^	58 (33.5)^*∗*^
Hypertension, *n* (%)	112 (38)	58 (49.2)^*∗*^	54 (31.2)^*∗*^

Oxygen requirement *n* (%)			
No use	5 (1.7%)	----	5 (2.9%)
Nasal prongs	94 (32.3%)	30 (25.5%)	64 (37%)
Mask	133 (45.7%)	49 (41.5%)	84 (48.6%)
High flow	42 (14.4%)	28 (23.7%)	14 (8%)
CPAP	1 (0.3%)	----	1 (0.6)
IMV	16 (5.5%)	11 (9.3%)	5 (2.9)

Medications use			
Metformin, *n* (%)	72 (25)	38 (32.2)^*∗*^	34 (19.7)^*∗*^
ACEi or ARBs, *n* (%)	75 (26)	25 (21)	50 (29)
Diuretics, *n* (%)	51 (18)	44 (37)^*∗*^	7 (4)^*∗*^
Dexamethasone, *n* (%)	285 (97.9)	116 (98.3)	169 (98)

Evaluation of scales			
APACHE II (pts)	9.70 ± 5.43	12.7 ± 6.0^*∗*^	7.7 ± 3.9^*∗*^
SOFA (pts)	3.06 ± 2.40	4.1 ± 3.0^*∗*^	2.40 ± 1.50^*∗*^
Laboratory exams at admission			
Hemoglobin (gr/dL)	14.65 ± 2.30	14.4 ± 2.4	14.8 ± 2.2
Leukocytes (×10^9/L^)	11.16 ± 5.21	12.6 ± 5.58	10.0 ± 4.7
Lymphocytes (×10^9/L^)	9.73 ± 5.17	0.94 ± 0.52	1.0 ± 0.51
Platelets (×10^9/L^)	263 ± 89	266 ± 91	260 ± 87
Glucose (mg/dL)	183 ± 121	215 ± 151^*∗*^	161 ± 88^*∗*^
Hemoglobin A1c (%)	7.9 ± 2.5	8.18 ± 2.6	7.8 ± 2.4

Inflammatory markers at admission			
LDH (*μ*/L)	391 ± 169	428 ± 166	367 ± 168
Ferritin (*μ*g/L)	815 ± 529	884 ± 592	737 ± 486
D-dimer (mg/mL)	434 (170–1483)	670 (230–2706)^*∗*^	327 (148–927)^*∗*^
CRP (mg/dL)	148 ± 108	158 ± 110	142 ± 107
Procalcitonin (ng/mL)	0.28 (0.11–0.77)	0.72 (0.34–2.01)^*∗*^	0.16 (0.09–0.34)^*∗*^
Severe sepsis/Septic shock, *n* (%)	66 (23)	58 (49)^*∗*^	8 (4.6)^*∗*^
Creatinine, at baseline (mg/dL)	0.80 ± 0.19	0.87 ± 0.22	0.74 ± 0.15
Stages of AKI, *n* (%)	---	61(51.7%)	---
1	---	16 (13.6%)	---
2	---	41 (34.7%)	---
3			

Recovery from AKI, *n* (%)	---	65 (55)	---
Yes	---	53 (45)	---
No			

AKI: acute kidney injury; BMI: body mass index; CRP: C-reactive protein; LDH: lactate dehydrogenase; CPAP: continuous positive airway pressure; IMV: invasive mechanical ventilation. ACEi or ARBs: angiotensin-converting enzyme inhibitors or angiotensin receptor blockers. ^*∗*^*p* < 0.05, comparison between groups with and without AKI.

**Table 2:** Markers of inflammation on hospitalization and during follow-up in patients with COVID-19 and acute kidney injury (AKI).

	AKI total	AKI 1	AKI 2 and 3
	*N* = 118	*N* = 61	*N* = 57
*Ferritin*			
On hospitalization	884 ± 592	839 ± 479	933 ± 699
Day-3	815 ± 508	637 ± 392	964 ± 520
Day-6	846 ± 499	594 ± 423	1111 ± 439
Day-12	972 ± 510	781 ± 516	1104 ± 482

*LDH (μ/L)*			
On hospitalization	428 ± 166	396 ± 168	462 ± 158
Day-3	474 ± 391^§^	388 ± 249	578 ± 498
Day-6	383 ± 157	368 ± 132	416 ± 176
Day-12	339 ± 140	340 ± 153	339 ± 133

*D-dimer (ng/mL)*			
On hospitalization	670 (230–2706)	543 (210–1830)	970 (274–3260)
Day-3	1259 (472–4308)	921(239–3683)	2264 (855–2264)
Day-6	1727 (646–3911)	960 (588–2534)^*∗*^	2928 (868–5000)^*∗*^
Day-12	1782 (708–3201)	1007 (461–1940)	2234 (1702–4452)

*CRP (mg/dL)*			
On hospitalization	158 ± 110	148 ± 109	171 ± 112
Day-3	105 ± 100^§^	83 ± 86	125 ± 107
Day-6	85 ± 91	78 ± 110	91 ± 72
Day-12	115 ± 76	81 ± 76	143 ± 66

*Procalcitonin (ng/mL)*			
On hospitalization	0.72 (0.34–2.01)	0.53 (0.22–1.13)^*∗*^	1.0 (0.52–2.80)^*∗*^
Day-3	0.42(0.20–1.90)	0.28 (0.15–0.64)^*∗*^	1.13 (0.33–7.52)^*∗*^
Day-6	0.49 (0.16–1.56)	0.23 (0.11–.62)^*∗*^	1.29 (0.20–3.9)^*∗*^
Day-12	1.18 (0.37–2.96)	0.43 (0.15–1.16)^*∗*^	1.94 (0.56–6.10)^*∗*^

AKI: acute kidney injury; CRP: C-reactive protein; LDH: lactate dehydrogenase; ^§^*p* < 0.05, vs. no AKI ^*∗*^*p*=<0.05, AKI 1 vs. AKI 2 and 3.

**Table 3:** Multivariate analysis for risk factors associated with acute kidney injury (AKI) in patients with COVID-19.

Multivariate analysis		
Variable	RR	CI 95%	*p*
Phosphate	1.39	1.13–1.72	0.002
APACHE	2.0	1.17–3.40	0.011
Procalcitonin	2.09	1.26–3.50	0.005

## Data Availability

Most clinical data are restricted to protect information from misuse. The data may be shared by the OPD, Hospital Civil de Guadalajara, and Dr. Juan I Menchaca (*HCG-JIM*) after approval from the Ethics Committee of the Institution. The data supporting the findings of this study are available from the corresponding author upon request.

## Abbreviations

COVID-19: Coronavirus disease 2019
SAH: Systemic arterial hypertension
DM: Diabetes mellitus
AKI: Acute kidney injury
CRP: C-Reactive protein
PCT: Procalcitonin
LDH: Lactate dehydrogenase
ALT: Alanine aminotransferase
SCr: Serum creatinine
CRRT: Continuous renal replacement therapy
RT-PCR: Reverse transcriptase polymerase chain reaction
KDIGO: Kidney disease improving global outcomes
APACHE II: Acute physiology and chronic health evaluation II
SOFA: Sequential organ failure assessment
eGFR: Estimated glomerular filtration rate
CKD: Chronic kidney disease
PaO_2_/FiO_2_: Oxygen to fraction of inspired oxygen
BMI: Body mass index
IMV: Invasive mechanical ventilation
ACEi: Angiotensin-converting enzyme inhibitor
ARBs: Angiotensin receptor blockers
NGAL: Neutrophil gelatinase-associated lipocalin
KIM-1: Kidney injury molecule 1
IFN-g: Interferon-gamma.
